# The improvement effect of insoluble dietary fiber of *Polygonatum sibiricum* on hyperlipidemia in high-fat diet mice via gut microbiota and metabolites

**DOI:** 10.3389/fnut.2025.1601867

**Published:** 2025-08-04

**Authors:** Yanli Ma, Jingxuan Ke, Yuan Wang, Yuhui Zhou, Xinyu Gao, Xin Wang, Qingshan Shen

**Affiliations:** ^1^Zhang Zhongjing School of Chinese Medicine, Nanyang Institute of Technology, Nanyang, China; ^2^College of Food Science and Technology, Hebei Agricultural University, Baoding, China

**Keywords:** insoluble dietary fiber, *Polygonatum sibiricum*, antihyperlipidemic effect, gut microbiome, metabolite profile

## Abstract

**Objective:**

*Polygonatum sibiricum* is rich in insoluble dietary fiber (IDF), but its antihyperlipidemic effect remains unclear. This study investigated the antihyperlipidemic effect of *Polygonatum sibiricum*’s IDF (PIDF) in high-fat diet mice.

**Methods:**

Male C57BL/6 J mice were fed with a high-fat diet continuously for 8 weeks. At the same time, the low-dose and high-dose groups were supplemented with 0.5 g/kg·BW and 1.0 g/kg·BW of PIDF, respectively. The weight and food intake of the mice were measured during the experiment. After 8 weeks of feeding, the organ weight, serum indexes, and liver function were investigated. Furthermore, the mechanism of antihyperlipidemic was explained by analyzing the gut microbiota and metabolites.

**Results:**

The results of the LIDF and HIDF showed that the PIDF treatment significantly alleviated the liver and kidney weight and body fat index. PIDF administration remarkably increased the high-density lipoprotein cholesterol level and enhanced hepatic superoxide dismutase activity in high-fat diet-fed mice. The levels of total cholesterol, triglycerides, low-density lipoprotein cholesterol, glucose, and aspartate transaminase in the HIDF were significantly lower than in the high-fat diet group. In addition, PIDF supplements also decreased the ratio of *Bacillota* to *Bacteroidota*, increasing the relative abundance of *Alistipes* and *Akkermansia*. Furthermore, metabolites suggest that dietary increases in PIDF can promote lipid and amino acid metabolism. Hence, PIDF improves lipid metabolism by regulating the gut microbiome and influencing host metabolism.

**Conclusion:**

It can be concluded that PIDF may alleviate hyperlipidemia by regulating cholesterol metabolism, increasing the abundance of beneficial microorganisms, and controlling its metabolites. The results of this study accelerated the application of PIDF in the health food industry.

## 1 Introduction

With high-fat diets becoming the eating habit or hobby of most people, hyperlipidemia has become a global problem of chronic diseases that threaten human health. Animal experiments and pharmacological studies have shown that hyperlipidemia and obesity are closely related to gut microbiota (1, 2). The interaction between non-starch polysaccharides, especially plant dietary fiber and intestinal microorganisms, has become a new research focus (3, 4). Dietary fiber can improve gut microbes, clean the intestinal tract, and prevent intestinal diseases (5). According to its water solubility, dietary fiber can be divided into two categories: water-insoluble dietary fiber (IDF) and water-soluble dietary fiber (SDF). Among these, IDF can also increase satiety and exhibit other beneficial effects (6). Moreover, IDF cannot be digested by the body’s digestive enzymes and passes directly to the large intestine, where it is metabolized by microorganisms in the gut (7). Therefore, IDF also has significant efficacy in improving intestinal microbes (8). The balanced dietary guidelines for residents suggest that a plant-based diet can lead to higher dietary fiber intake, thereby improving human health (6). Natural plant dietary fiber possesses the characteristics of green health, is a vast source, and has a low cost, which makes it a considerable application prospect in developing functional foods for antihyperlipidemic purposes.

The hypolipidemic effect of dietary fiber from natural plants has been widely studied. Still, the mechanism is different, and it is typically the result of the combined action of several mechanisms. Reports from animal experiments indicated that plant dietary fiber can play a role in the hypolipidemic effect by regulating oxidative stress and lipid metabolism. The mechanism by which plant dietary fiber improves hyperlipidemia is as follows: (1) regulating triglyceride and fatty acid metabolism. The study of Wang et al. (9) demonstrated that soybean IDF can inhibit the expression of SREBP1c and SCD1 mRNA in hyperlipidemia liver, thereby reducing TC and TG levels, and play a beneficial role in improving HFD-induced dyslipidemia and liver steatosis in mice. (2) Regulate cholesterol metabolism. Liu et al. (10) found that dietary fibers from bergamot could inhibit the increase of LDL-C in the blood, promote the transport and consumption of oil in the liver, and subsequently reduce the levels of cholesterol and lipids in the body, demonstrating a noticeable hypolipidemic effect. (3) Regulate gut microbes. Zhang et al. (11) reported that after 8 weeks of intervention with a high-fat diet in mice, those fed an IDF diet extracted from brown seaweed *Laminaria japonica* showed a significant improvement in intestinal microbial composition, primarily by substantially increasing the relative abundance of *Akkermansia*. Additionally, biochemical results showed that serum cholesterol concentration and glucose level were significantly reduced. Another study also showed that insoluble dietary fibers and total dietary fiber obtained from *Caulerpa lentillifera* exhibited increased levels of acetic acid and propionic acid in metabolites in the supplemental dietary fiber groups compared to the high-fat diet group (12). It has been reported that under the fermentation of gut microbiota, dietary fiber produces more short-chain fatty acids, which have a noticeable effect on improving metabolic syndrome, especially hyperlipidemia (3).

*Polygonatum sibiricum* is one of the homologous resources of traditional medicine and food, which has essential edibility and therapeutic value. Except for polysaccharides and saponins, *Polygonatum sibiricum* is rich in IDF, with a content of up to 88% (13). Currently, research on *Polygonatum sibiricum* primarily focuses on identifying functional ingredients, including polysaccharides and saponins (5). A large amount of residue is generated after the extraction of active ingredients and is not fully utilized. The *Polygonatum sibiricum* residue contains a large amount of dietary fiber, which has potential applications in the food industry (13).

In our previously published report, PIDF was the high purity (76.7%) (14). Additionally, *in vitro* studies have demonstrated that PIDF is more effective in adsorbing cholesterol (35.95 mg/g) and glucose (24.63 μmoL/g) (15). However, there is no report on the actual hypolipidemic effect of PIDF *in vivo* experiments, and its mechanism remains largely unrecognized. Furthermore, this study highlighted its anti-hyperlipidemic effect and modulation of gut microbiota in high-fat-fed mice. Therefore, this study investigated the mechanism by which gut microbiome regulates anti-hyperlipidemia. In addition, we also investigated the regulatory effect of PIDF on caecum metabolites to further elucidate the mechanism of its hypolipidemic action. This study will provide valuable theoretical evidence for applying PIDF as a food for alleviating hyperlipidemia.

## 2 Materials and methods

### 2.1 *Polygonatum sibiricum* insoluble dietary fiber

Simply, *Polygonatum sibiricum* residue powder was enzymatic hydrolysis by *α*-amylase (0.25%) at pH 6, 60°C for 1 h. Next, the mixture was enzymatically hydrolyzed by papain (0.125%) at pH 5, 50°C for 1 h. After enzymolysis, the mixture was subjected to ultrasonic treatment (Ningbo Xinzhi Biotechnology Co., Ltd., Ningbo, China) at 50°C for 20 min (ultrasonic 2 s and intermittent 2 s). Then, collect the residue after centrifuging (2,683 g for 10 min) the mixture. Finally, the residue was washed twice with water and once with 70% ethanol; the residue was ball-milled (CJM-SY-B, Qinhuangdao Bomao New Material Technology Co., Ltd., Qinhuangdao, China) at 380 rpm for 8 h to obtain a consistent particle size distribution, and the processed powders were designated as PIDF. The PIDF has a consistent purity (76.7%) and consists mainly of cellulose (24.4%), hemicellulose (31.5%), lignin (20.8%), protein (7.5%), starch (4.1%), and ash (0.97%) (14). According to our report (15), PIDF exhibited better functional properties: water-holding capacity (4.36 g/g), oil-holding capacity (2.62 g/g), water-swelling capacity (2.40 mL/g), ion exchange capacity (0.27 mmol/g), and cholesterol (35.95 mg/g) and glucose (24.63 μmoL/g) adsorption capacity. The particle size distribution and zeta-potential of PIDF were 9.96 μm and −25.95 mV, respectively (15).

### 2.2 Animal experiment

The ethics committee for experimental animals of Nanyang Institute of Technology reviewed all the animal experiments (Animal Experiment Ethics Review No. [2024]001). Here, seven-week-old male C57BL/6 J mice (SPF grade, 18 ± 2 g) were purchased from Beijing Vital River Laboratory Animal Technology Co., Ltd. (Beijing, China). During the adaptation feeding period (7 days), all mice were fed with a standard diet. All mice had free access to water and food. The feeding temperature was maintained at 22 ± 2°C, with a relative humidity of 55 ± 10%, and a 12 h light–dark cycle. The obesity model was established according to Wang et al. (9) and Zhang et al. (11). After adaptive feeding, the mice (eight-week-old male) were randomly divided into 4 groups, with 8 animals in each group and 4 animals in each cage. If the data of 1–2 animals were outliers, the data of that mouse were excluded. The normal control group (NC) was fed a standard diet. The high-fat diet control group (HC) was fed a high-fat diet (60% kcal fat: protein 26%, carbohydrate 26%, fat 35%). According to the report (9), the low-dose group (LIDF) was fed with a high-fat diet supplemented with gavage administration of 0.5 g per kg·BW of PIDF per mouse per day, and the high-dose group (HIDF) was fed a high-fat diet supplemented with gavage administration of 1.0 g per kg·BW of PIDF per mouse per day. The mice were fed a PIDF-supplemented diet for 8 weeks.

### 2.3 Body weight and tissues collected

The weight of each mouse in each group was recorded weekly. The food intake of each cage was recorded every day. The total amount of food divided by the number of mice in the cage gives the daily food intake for each mouse. At the end of the 8-week experiment, the abdomen of the mouse was gently pressed to stimulate defecation. After the mouse defecated, the fresh fecal samples were quickly collected into a sterile test tube. Mice were anesthetized with isoflurane after a 12 h fast (Jiangsu Hengfengqiang Biotechnology Co., LTD., Jiangsu, China). Blood was collected through the orbital venous plexus, centrifuged at 4°C, and serum was obtained by spinning at 3500 rpm for 15 min. The serum was stored at −80°C for biochemical analysis.

After euthanasia, the kidneys, spleen, liver, and adipose tissue of the mice (perirenal and epididymis) were dissected and weighed. At the same time, cecal contents were collected. The organ index was measured by calculating the ratio of organ weight to body weight. Divide the liver into two parts, wrap one in tin foil, and freeze at −80°C. The other part of the liver, perirenal fat, and epididymal fat were soaked with 4% paraformaldehyde at room temperature.

### 2.4 Serum biochemical analysis

Serum lipid parameters, including total cholesterol (TC), triglycerides (TG), glucose (GLU), aspartate transaminase (AST), alanine aminotransferase (ALT), high-density lipoprotein cholesterol (HDL-C), and low-density lipoprotein cholesterol (LDL-C) were measured using commercial kits (Shandong Biobase Industry Co., LTD, Jinan, China) per the manufacturer’s instructions.

### 2.5 Histological analysis

Liver and epididymal fats fixed in 4% paraformaldehyde were embedded and sectioned into 4 μm sections. The slices were stained with hematoxylin–eosin (H&E) (12). Then, it was viewed under a light microscope (Olympus, Japan) and photographed. Additionally, the Nonalcoholic Fatty Liver Disease (NAFLD) activity score of liver slices was evaluated. The detailed scoring criteria refer to the guidelines of the National Institute of Health’s NASH Clinical Research Pathology Working Group of the United States (16).

### 2.6 Biochemical indexes of liver tissue

The activities of glutathione peroxidase (GSH-PX) and superoxide dismutase (SOD), malondialdehyde (MDA) content, and total antioxidant capacity (T-AOC) of liver tissue were determined according to the instructions of special kits (Nanjing Jiancheng Biotechnology Company, Nanjing, China).

### 2.7 Gut microbiota analysis

Mouse feces were collected to isolate total DNA, and the V3–V4 region of the 16S rRNA gene was amplified using PCR with forward primer 341F (CCTAYGGGRBGCASCAG) and reverse primer 806R (GGACTACNNGGGTATCTAAT). Overlapping paired-end reads were merged using fast-join and processed with QIIME. Illumina readings with an average score more excellent than 20 were selected for further analysis (8). The 16S rRNA gene sequence was used to classify the operational taxon units (OTUs) of the detected bacteria. Alpha diversity analysis was performed for the classified microorganisms, and Chao, Pielou’s, Shannon, and Simpson indices were used to characterize microbial diversity and richness. The Beta diversity of the classified microorganisms was analyzed, and a Venn diagram represented the intersection size of OTUs between different groups. Meanwhile, the similarity of microorganisms between various groups was illustrated using principal component analysis (PCA) (17).

### 2.8 Non-targeted metabolite analysis

Metabolomics analysis was performed using ultra-high-performance liquid chromatography (LC) tandem mass spectrometry (MS/MS). Briefly, the cecum was frozen with liquid nitrogen. Take a 100 mg tissue sample into an EP test tube, then add 500 μL of an 80% methanol solution. Next, the mixture was swirled over an ice bath for 5 min until well mixed. The mixture is then centrifuged at 15000 g, 4°C for 20 min. Collect the supernatant and add mass spectrometry water to adjust the methanol concentration of the supernatant to 53%. Then, the mixture was centrifuged at 15000 g, 4°C for 20 min. Finally, the supernatant was analyzed by LC–MS/MS.

LC was performed using the Vanquish UHPLC System (Thermo Fisher Scientific, Germany) and a Hypersil Gold column (100 × 2.1 mm, 1.9 μm) (Thermo Fisher Scientific, United States); the column temperature was maintained at 40°C. The flow rate was set at 0.2 mL/min. The mobile phases consisted of 0.1% formic acid (v/v) (A) and methanol (B). The gradient elution program was as follows: 0–1.5 min, 98% A; 1.5–3 min, 98–15% A; 3–10 min, 15–0% A, 10–10.1 min, 0–98% A, 10.1–12 min, 98% A. ESI (+) and ESI (−) were analyzed by Q Exactive™ HF/Q Exactive™ HF-X MS/MS (Thermo Fisher Scientific, Germany) with an ESI ion source, respectively. The parameters were set as follows: Spray Voltage: 3.5 kV, sheath gas pressure: 35 psi; aux gas flow:10 mL/min; capillary Temp: 320°C, S-lens RF level: 60, Aux gas heater temp:350°C, MS/MS scans: data-dependent scans, MS1 range: m/z 100–1,500 (18). All multivariate data analyses and modeling were performed using the NovoMagic Platform.^1^ After scaling the data, the models were constructed using principal component analysis (PCA) and partial least squares discriminant analysis (PLS-DA).

### 2.9 Statistical analysis

Statistical analysis was performed with SPSS 27.0 software (IBM Corporation, NY, United States) and Origin 2024 software (OriginLab, UAS). Results were presented as means ± standard deviation. Differences in experimental data were analyzed by one-way analysis of variance (ANOVA) with Duncan’s test. The statistically significant results were considered as *p* < 0.05, *n* = 6.

## 3 Results

### 3.1 PIDF alleviates body weight

**Figure 1:**
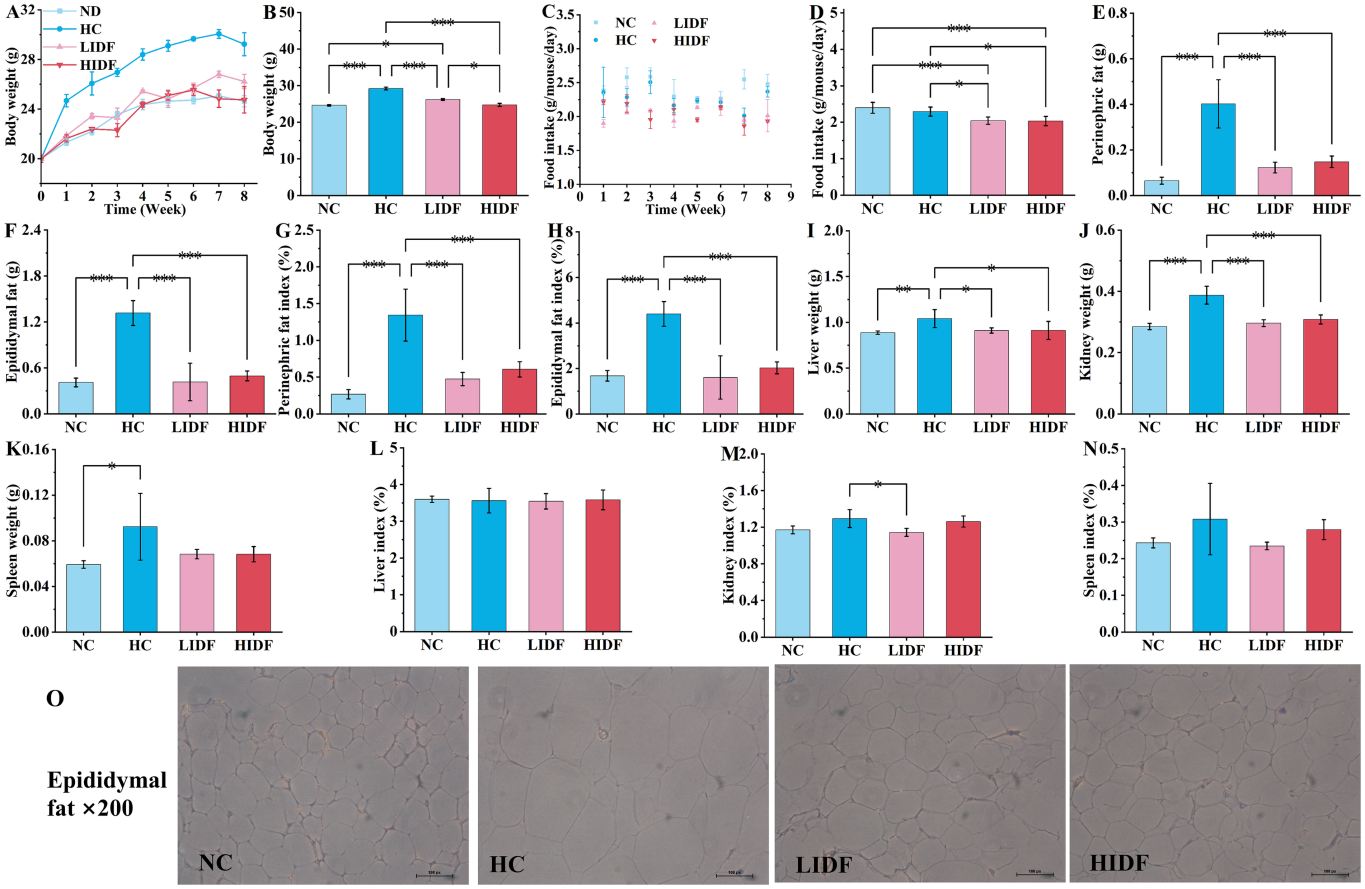
Effects of PIDF on high-fat diet mice. **(A)** Body weight of every week; **(B)** Body weight at the end of the experiment; **(C)** Food intake during 8 weeks; **(D)** The average food intake of each mouse; **(E)** Perinephric fat; **(F)** Epididymal fat; **(G)** Perinephric fat indexes; **(H)** Epididymal fat indexes; **(I)** Liver weight; **(J)** Kidney weight; **(K)** Spleen weight; **(L)** Liver indexes; **(M)** Kidney indexes; **(N)** Spleen indexes; **(O)** H&E staining of epididymal fat. Data presented as mean SEM, *n* = 6, * *p* < 0.05, ** *p* < 0.01, *** *p* < 0.001.

Figure 1A illustrates the weekly weight change curve for the four groups of mice over 8 weeks. The weight of mice is a direct indicator of their health, especially whether they are overweight or obese (19). During the experimental feeding period, the weight of each group of mice increased with age. Compared with the NC group, the weight gain trend in the HC group was significantly accelerated after the second week (*p* < 0.05). It is worth mentioning that at the end of the experiment, the weight of the mice in the HC group (29.23 g ± 0.92 g) exceeded that of the NC group (24.63 g ± 0.45 g) by more than 15%, indicating that the obese mice had been successfully modeled. As depicted in Figure 1A, the weight gain rate of mice in the LIDF and HIDF groups was significantly reduced after PIDF supplementation, and this trend became more pronounced as the mice aged.

Figure 1B shows the weight of the four groups of mice at the end of the experiment. As expected, the body weight of mice in the LIDF (26.22 g ± 0.57 g) and HIDH (24.75 g ± 0.45 g) groups was significantly lower than that in the HC group (*p* < 0.001) (Figure 1B). The body weight of mice in the HIDF group was the same as that in the NC group, with no significant difference (*p* > 0.05) (Figure 1B). In addition, compared with the LIDF group, high doses of PIDF supplementation (the HIDF group) resulted in more significant weight loss in mice (*p* < 0.05) (Figure 1B). The results showed that the dietary addition of PIDF could conspicuously slow the weight gain of mice on a high-fat diet (20).

### 3.2 Effect of PIDF on food intake in high-fat diet mice

Figure 1C illustrates the trends in the average food intake of each mouse over the 8-week experiment period. Figure 1D compares the average food intake of each mouse in different groups throughout the experiment. The results showed that, without the PIDF dietary supplement, the food intake of the HC group (2.29 g/mouse/day) mice was significantly higher than that in the LIDF (2.04 g/mouse/day) and HIDF (2.03 g/mouse/day) groups (*p* < 0.05). Furthermore, the food intake of the LIDF and HIDF groups was considerably lower than that of the NC group (2.39 g/mouse/day) (*p* < 0.001), possibly due to the satiety induced by PIDF intake in the LIDF and HIDF groups.

### 3.3 The effect on the body fat index of mice

The effects of dietary supplementation of IDF on epididymal fat and perinephric fat accumulation in mice are described in Figures 1E,F. It was observed that at the end of the experiment, after 8 weeks of high-fat diet feeding, the fat accumulation in the HC group was excessive. Compared with the NC group (0.065 g ± 0.02 g, 0.41 g ± 0.06 g, respectively), the perinephric fat accumulation in the HC group increased by 83.8% (*p* < 0.001), and epididymal fat accumulation increased by 68.9% (*p* < 0.001). While PIDF significantly inhibited fat accumulation in the LIDF and HIDF groups (*p* < 0.001, Figures 1E,F) compared to the HC group, which ranged from 62.3 to 69.5%. Similarly, as shown in Figures 1G,H, the perinephric and epididymal fat indexes in the LIDF (0.47% ± 0.09, 1.61% ± 0.94%, respectively, *p* < 0.001) and HIDF (0.61% ± 0.10, 2.02% ± 0.26%, respectively, *p* < 0.001) groups were remarkably lower than in the HC group. No significant difference was observed in the effect of PIDF on fat accumulation in mice between the LIDF and HIDF groups, indicating that PIDF has a beneficial impact in inhibiting body fat accumulation. Meanwhile, as shown in Figure 1O, the adipocytes in the HC group were the largest, while those in the LIDF and HIDF groups were significantly reduced, indicating that PIDF decreased the lipid content of adipocytes (21). Overall, these results showed that PIDF treatment significantly alleviated the weight of perinephric and epididymal fat induced by a high-fat diet.

### 3.4 The effect on organ indices of mice

**Figure 2:**
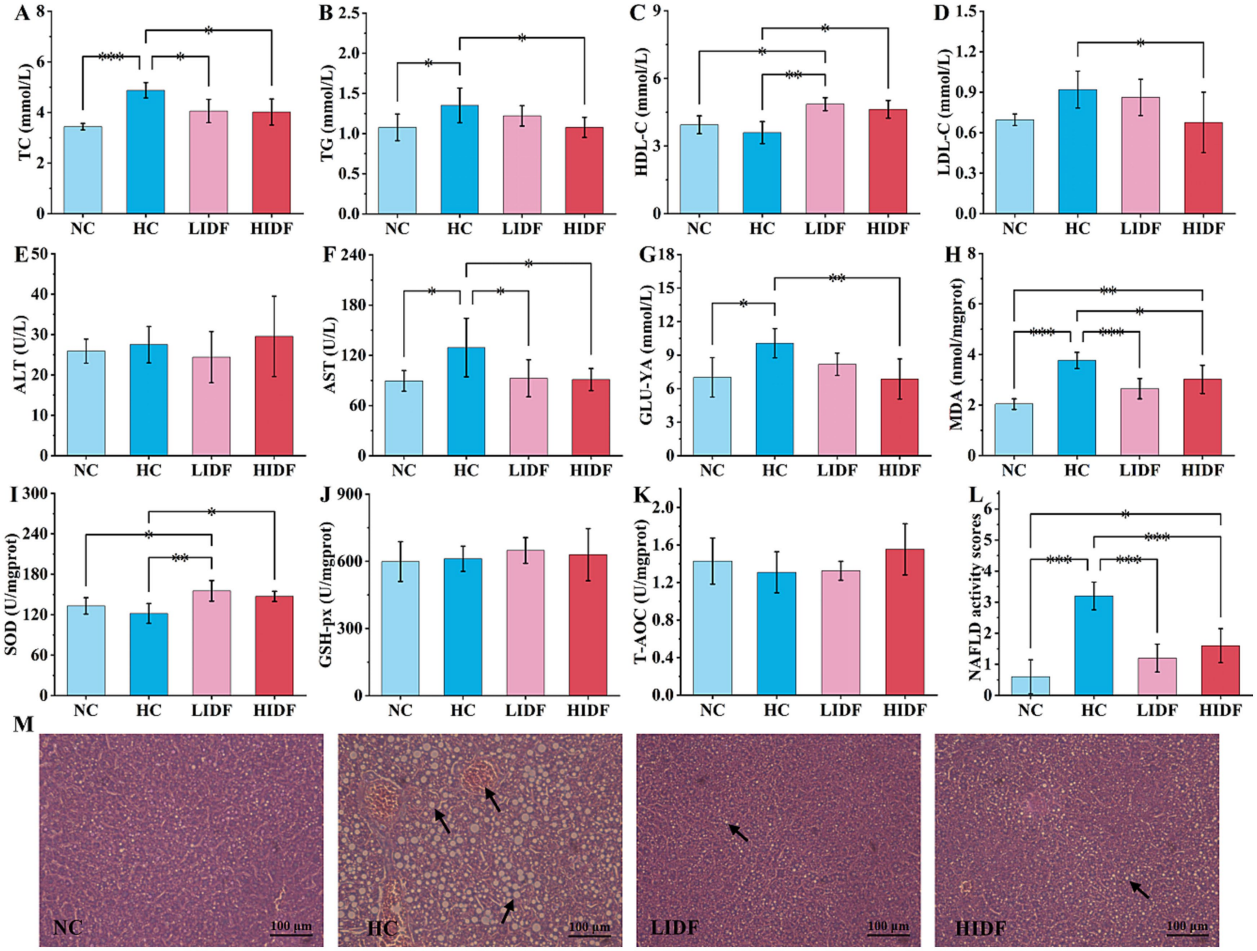
Effects of PIDF on blood lipid levels and activity of antioxidant enzymes in high-fat diet mice. **(A)** TC; **(B)** TG; **(C)** HDL-C; **(D)** LDL-C; **(E)** ALT; **(F)** AST; **(G)** GLU-YA; **(H)** MDA; **(I)** SOD; **(J)** GSH-Px; **(K)** T-AOC; **(L)** NAFLD activity score of the livers. **(M)** H&E staining of the liver (The arrows in images indicate hepatocyte fatty degeneration). Data presented as mean SEM, *n* = 6, * *p* < 0.05, ** *p* < 0.01, *** *p* < 0.001.

The effects of PIDF on organ weight and organ index in mice were observed in Figures 1I–N. After 8 weeks of high-fat diet feeding, the weight of the liver (1.0403 g ± 0.00986 g, *p* < 0.01), kidney (0.3875 g ± 0.0293 g, *p* < 0.001), and spleen (0.0925 g ± 0.0216 g, *p* < 0.05) in the HC group was the heaviest, which was significantly higher than that in the NC group (0.88763 g ± 0.0179 g, 0.2853 g ± 0.0104 g, 0.0525 g ± 0.0033 g, respectively). After the addition of PIDF, the organ weight of high-fat diet mice in the LIDF and HIDF groups was significantly reduced, especially the weight of the liver (0.9108 g ± 0.0285 g, 0.9120 g ± 0.098 g, respectively, *p* < 0.05, Figure 1I) and kidney (0.2960 g ± 0.111 g, 0.3082 g ± 0.0147 g, respectively, *p* < 0.001, Figure 1J) was markedly lower than that in the HC group. In addition, spleen weight was also lower in the PIDF dietary intervention groups compared to the HC group, although the difference was not significant (*p* > 0.05) (Figure 2K). The more fat it contains, the more its function is affected. As shown in Figures 1L–N, the kidney index in the LIDF group (1.1437% ± 0.0432%) was significantly decreased compared with the HC group (1.2942% ± 0.0976%, *p* < 0.05). However, there were no significant differences in liver and spleen indexes between the LIDF group and the HIDF group (*p* > 0.05).

### 3.5 The effect on the blood lipid level of mice

To investigate the alleviating effect of PIDF on hyperlipidemia in mice, the serum lipid indexes of mice were measured, and the results are shown in Figures 2A–G. It could be seen from the second column set, the level of TC (4.88 mmol/L ± 0.30 mmol/L, Figure 2A) and TG (1.35 mmol/L ± 0.22 mmol/L, Figure 2B), LDL-C (0.92 mmol/L ± 0.14 mmol/L, Figure 2D), AST (129.24 U/L ± 34.93 U/L, Figure 2F), and GLU-YA (10.07 mmol/L ± 1.30 mmol/L, Figure 2G) in the HC group mice significantly increased by 41.78% (*p* < 0.001), 25.44% (*p* < 0.05), 32.18% (*p* < 0.05), 44.54% (*p* < 0.05), and 43.49% (*p* < 0.05), respectively, and HDL-C decreased by 8.78% (3.59 mmol/L ± 0.49 mmol/L, Figure 2C) compared with the NC group, which indicated that the long-term feeding of high-fat diet resulted in dyslipidemia in mice (10, 22). Compared with the HC group, the contents of TC (4.05 mmol/L ± 0.46 mmol/L and 4.02 mmol/L ± 0.52 mmol/L, respectively, Figure 2A) and AST (92.61 U/L ± 22.3 U/L, 91.01 U/L ± 13.18 U/L, respectively, Figure 2F) in the mice’s serum of the LIDF and HIDF groups were significantly ameliorated (*p* < 0.01), and the content levels were similar to those in the NC group (*p* > 0.05). At the same time, there was no significant difference in the ALT content in the blood of mice in the NC, HC, LIDF, and HIDF groups (*p* > 0.05).

However, when high doses of PIDF were given (HIDF group), the TG, LDL-C, and GUL-YA levels in the serum of the mice were significantly lower than those in the HC group, with content of (1.08 ± 0.13) mmol/L, (0.68 ± 0.22) mmol/L, and (6.87 ± 1.80) mmol/L, respectively (*p* < 0.05). The findings of this study are consistent with those reported in previous research by Isken et al. (23) and Fang et al. (24). In addition, when supplementing a low dose of PIDF, there was no significant effect on these three serum biochemical indexes between the LIDF group and the HC mice (*p* > 0.05). It is worth mentioning that compared with the HC group, PIDF supplementation significantly increased the serum HDL-C content in the LIDF and HIDF groups by 35.08% (*p* < 0.01) and 21.30% (*p* < 0.05), respectively (Figure 2C). HDL-C is considered the “good” cholesterol because of its ability to transport cholesterol from peripheral tissues back to the liver for metabolism, helping to reduce the risk of cardiovascular disease (25, 26). These results indicated that a specific dose of PIDF can significantly reduce the serum TC, TG, and LDL-C levels and increase HDL-C level in hyperlipidemia animal models, which improves blood lipid health.

### 3.6 Activity of antioxidant enzymes in the liver of mice

The detected indicators of oxidative stress are described in Figures 2H–K. The content of MDA in the NC group was the lowest (2.03 nmol/mgprot ± 0.20 nmol/mgprot, *p* < 0.001), indicating that a regular diet would not cause abnormal liver metabolism (Figure 2H) (27). As shown in Figure 2H, MDA content in the HC group mice (3.76 nmol/mgprot ± 0.32 nmol/mgprot) was significantly higher than in the other three groups, indicating that a high-fat diet caused certain liver damage. After the PIDF diet intervention, MDA levels decreased in both the high-dose group (2.68 nmol/mgprot ± 0.40 nmol/mgprot, *p* < 0.01) and the low-dose group (3.02 nmol/mgprot ± 0.56 nmol/mgprot, *p* < 0.001), with proportions of 19.80 and 29.68%, respectively. The results showed that PIDF had a protective effect on the liver, preventing liver damage caused by a high-fat diet. As expected, the high-fat diet reduced SOD activity (121.83 U/mgprot ± 14.72 U/mgprot) in the mice’s liver compared to the NC group (133.06 U/mgprot ± 12.07 U/mgprot), resulting in oxidative stress. The SOD activity in LIDF and HIDF groups was increased by 27.51% (155.35 U/mgprot ± 15.25 U/mgprot, *p* < 0.01) and 20.81% (147.18 U/mgprot ± 7.51 U/mgprot, *p* < 0.05), respectively (Figure 2I). The results showed that the PIDF dietary intervention could effectively improve the oxidative stress caused by a high-fat diet. Simultaneously, there was no detectable increase in GSH-Px and T-AOC activity in the LIDF and HIDF groups (Figures 2J,K, *p* > 0.05). The results suggest that PIDF intervention may help alleviate liver disease caused by a high-fat diet.

The liver is a vital organ in the human body, and a prolonged high-fat diet can lead to excessive fat accumulation in the liver, ultimately causing liver damage. Therefore, to demonstrate that the PIDF diet can improve this phenomenon, the livers of four groups of mice were sliced and stained with H&E to observe the accumulation of fat in the liver more clearly (Figure 2M). The results showed that the liver cells of mice on a standard diet had normal morphology, clear boundaries, and a neat arrangement, with no fat accumulation observed (17). However, hepatocytes in the HC group were severely damaged, mainly due to the accumulation of a large number of fat particles within hepatocytes, which crowded out the nucleus to the edge, with the possibility of lipopathy (28). This indicated that a high-fat diet can seriously damage the liver, resulting in a considerable accumulation of fat in the liver and affecting liver function. On the contrary, the form of liver cells in the PIDF supplementation mice was as expected. Additionally, the fat accumulation in the livers of mice in the LIDF and HIDF groups was reduced, which slowed the damage to liver cells caused by a high-fat diet (29). Furthermore, the NAFLD activity score of the livers in the four groups was assessed (Figure 2L). The results indicated that the NAFLD activity score of the liver in the HC group was 3.2 ± 0.45, and extremely significantly higher than that of the other three groups (*p* < 0.001). Hence, the livers of mice in the HC group were identified as NAFLD. At the same time, the NAFLD activity scores of the livers of mice in the NC, LIDF, and HIDF groups were less than 2, which could exclude the possibility of NAFLD (30). In conclusion, these findings demonstrated that PIDF supplementation successfully ameliorated lipid metabolic disorders in mouse livers caused by long-term high-calorie diets.

### 3.7 The effects on the gut microbiota of mice

**Figure 3:**
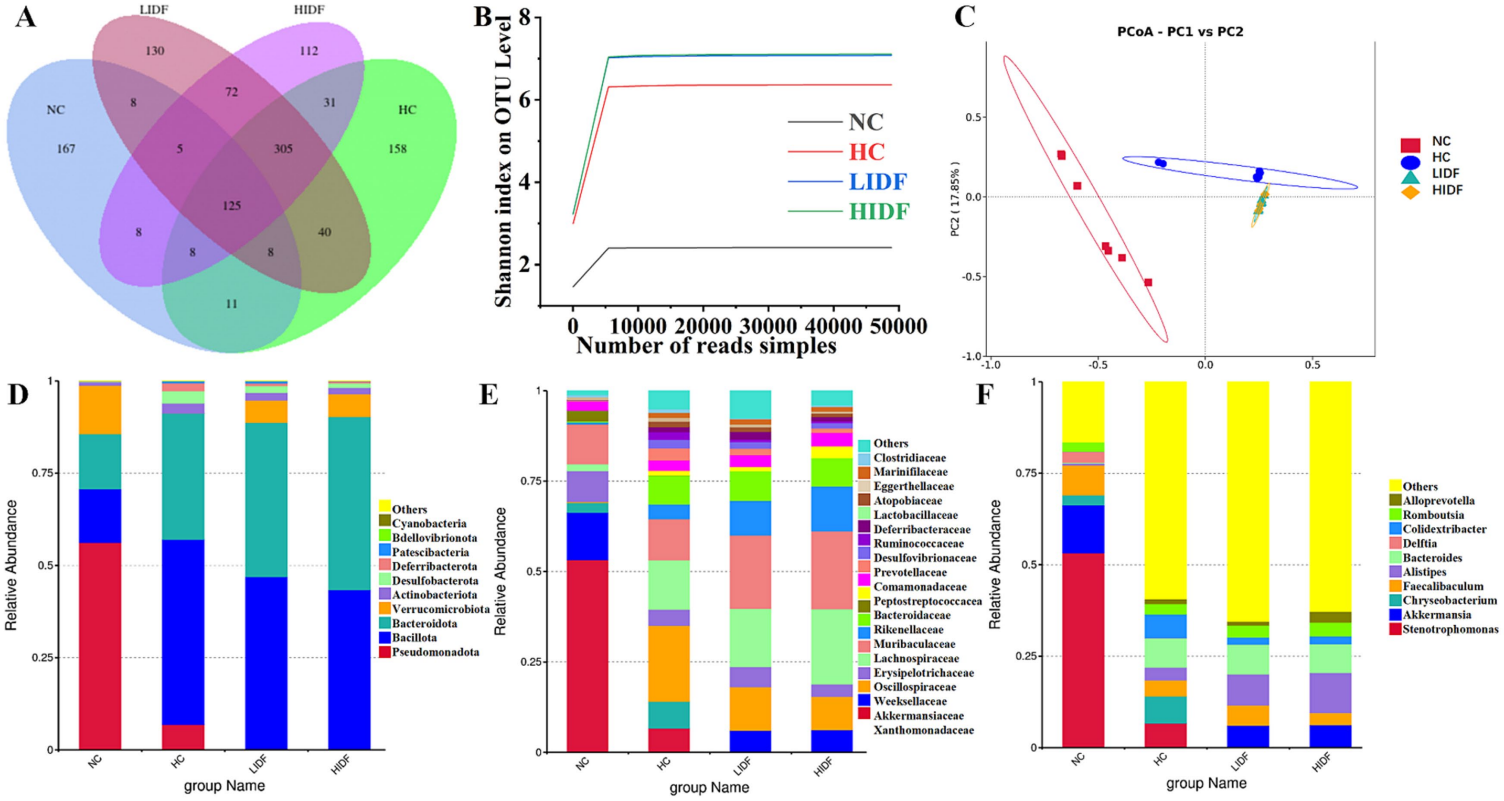
Effects of PIDF on gut microbiota in high-fat diet mice. **(A)** Venn diagram; **(B)** Dilution curve and species accumulation curve of each group; **(C)** PCoA; **(D)** Gut microbial composition at the phylum levels; **(E)** Gut microbial composition at the family levels; **(F)** Gut microbial composition at the genus levels.

In this study, the regulatory effect of PIDF on intestinal microbes was investigated. Firstly, the Alpha diversity index of mouse gut microbiota was analyzed, and the results are shown in Table 1. The results showed that the Chao1, Shannon, and Simpson indices of the HIDF and LIDF groups were increased with dietary fiber intake. These results suggested that IDF could increase the richness and diversity of gut microbiota. On this basis, a Venn diagram based on operational taxon (OTUs) was used to analyze the similarity of gut microbiota among different groups. According to the Venn diagram of the four groups of mice (Figure 3A), 348, 686, 666, and 693 OTUs were detected in the NC, HC, LIDF, and HIDF groups, respectively. The four unique sections were 167, 158, 112, and 130 OTUs, respectively. The OTUs shared by the HC, LIDF, and HIDF groups with the NC group were 152, 146, and 146, respectively. The OTUs shared by the LIDF and HIDF groups with the HC group were 378 and 469, respectively. These results suggest that PIDF may play a role in regulating the intestinal microbial structure of mice on a high-fat diet (17). The dilution curve and species accumulation curve for each group of samples are shown in Figure 3B. At the initial sequencing stage, the curve exhibited a rapidly rising trend, and as the sequencing quantity increased, the curve for each group gradually leveled off. After further increasing the sequencing volume, only a few new OTUs were produced, indicating that the sequencing has reached saturation. These results suggest that the amount of sequencing data has fully covered the microbial diversity in the samples.

**Table 1:** Alpha diversity analysis.

Groups	Chao1	Pielou’s	Shannon	Simpson
NC	175.2 ± 26.80***	0.50 ± 0.01***	3.65 ± 0.20***	0.83 ± 0.02***
HC	427.46 ± 25.09	0.75 ± 0.09	5.71 ± 1.40	0.91 ± 0.08
LIDF	493.29 ± 11.58*	0.80 ± 0.01	7.14 ± 0.06***	0.99 ± 0.00**
HIDF	501.59 ± 19.44**	0.80 ± 0.01	7.17 ± 0.12***	0.98 ± 0.00**

* Significant difference compared to the HC group. * *p* < 0.05; ** *p* < 0.01; and *** *p* < 0.001.

Additionally, *β* diversity analysis revealed the differences between the gut microbial communities of various mouse groups (31). Then, principal coordinate analysis (PCoA) was used to investigate the overall differences in the composition of intestinal bacteria in the four groups. Figure 3C showed that the four groups of microbial communities were distributed in different regions, and each group formed relatively close clusters. In particular, the clusters of the LIDF and HIDF groups with PIDF intervention were significantly different from those of the HC group. The results showed that a dietary increase in PIDF altered the intestinal microbial composition of mice fed a high-fat diet. The results were consistent with the report by Liu et al. (32).

**Figure 4:**
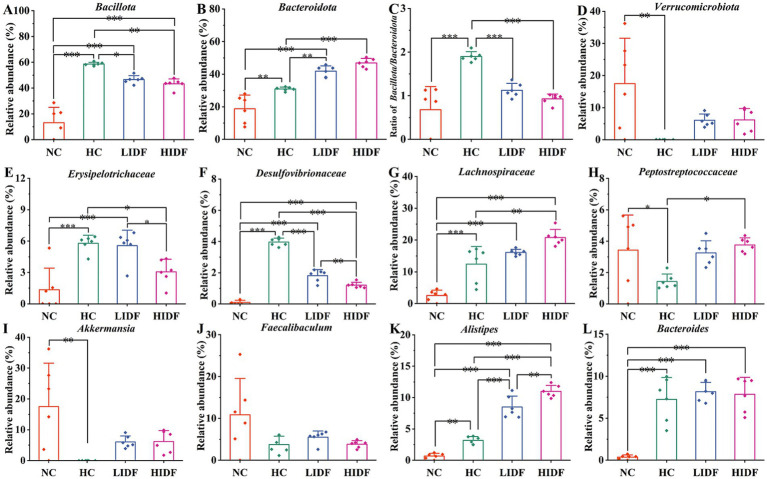
Relative abundances of **(A)** *Bacillota* and **(B)** *Bacteroidota*; **(C)** *Bacillota*/*Bacteroidota* ratio; **(D)** Relative abundances of *Verrucomicrobiota*; **(E)** *Erysipelotrichaceae*; **(F)** *Desulfovibrionaceae*; **(G)** *Lachnospiraceae*; **(H)** *Peptostreptococcaceae*; **(I)** *Akkermansia*; **(J)** *Faecalibaculum*; **(K)** *Alistipes*; **(L)** *Bacteroides.* Data presented as mean SEM, *n* = 6, * *p* < 0.05, ** *p* < 0.01, *** *p* < 0.001.

Further, we determined the composition of gut microbes in four groups of mice at the phylum, family, and genus levels to explore the effects of PIDF on microbial species and their relative abundance. The differences in gut microbiome composition at the phylum level among the four groups of mice are illustrated in Figure 3D. For the NC group, the dominant phyla are *p_Pseudomonadota*, *p_Bacillota*, *p_Bacteroidota*, and *p_Verrucomicrobiota*. At the same time, *Bacillota* and *Bacteroidota* are the dominant phyla in the other three groups. Figures 4A–D illustrates the changes in the relative abundance of specific bacteria. Compared with the NC group, the relative abundances of *p_Bacillota* (*p* < 0.001) and *p_Bacteroidota* (*p* < 0.01) in the HC group were significantly increased, resulting in a significant increase in the ratio of *Bacillota* to *Bacteroidota* (1.89 ± 0.11). In addition, the relative abundance of *p_Verrucomicrobiota* was significantly decreased in the HC group (*p* < 0.001). Multiple studies have shown that metabolic syndrome is associated with an increased ratio of *Bacillota* to *Bacteroidota* (31). This also suggested that the mice in the HC group had abnormal lipid metabolism. As can be seen in Figure 4A, PIDF intervention resulted in a considerable reduction in the abundance of *p_Bacillota*, from 58.65% ± 1.31% (HC group) to 46.59% ± 3.07% in the LIDF group (*p* < 0.05) and 43.27% ± 3.81% in the HIDF group (*p* < 0.01). On the contrary, compared to the HC group (30.93 ± 1.17%), the abundance of *p_Bacteroidota* exhibited a significant increase in the LIDF (41.83 ± 3.26%, *p* < 0.01) and HIDF (46.92 ± 2.75%, *p* < 0.001) groups. As a result (Figure 4C), the *Bacillota* to *Bacteroidota* ratio of the LIDF (1.12 ± 0.15) and HIDF (0.92 ± 0.11) groups was significantly decreased, which is similar to levels in NC mice (0.67 ± 0.53) (*p* > 0.05). These results suggest that PIDF can regulate gut microbiome and metabolic function and improve hyperlipidemia induced by a high-fat diet in mice. The study results of Yang et al. (33) showed that flaxseed polysaccharide also regulates the composition of intestinal microbes in high-fat diet mice by reducing the *Bacillota* to *Bacteroidota* ratio, thereby exerting an effect on decreasing serum lipids. In addition, as shown in Figure 4D, PIDF supplementation successfully increased the relative abundance of *p_Verrucomicrobiota* from 0.03% in the HC group to 6.05% ± 1.94% in the LIDF group and 6.18% ± 3.53% in the HIDF group.

At the family level (Figure 3E), compared with the NC group, high-fat diet feeding increased the relative abundance of these two types of harmful bacteria (*f_Erysipelotrichaceae* and *f_Desulfovibrionaceae*) in the HC group (*p* < 0.001, Figures 4E,F). In contrast, this trend was significantly suppressed after the daily addition of the PIDF diet, especially in the high-dose group (*p* < 0.001, Figures 4E,F). As shown in Figure 4G, the relative abundance of *f_Lachnospiraceae* in the HIDF and LIDF groups was higher than in the HC group. Studies have shown that low fermentable polysaccharides can increase the relative abundance of *f_Lachnospiraceae* in the gut (34). The growth of *f_Peptostreptococcaceae* in the intestines of high-fat diet mice was significantly inhibited, and the PIDF diet significantly increased the relative abundance of this bacteria, the level of which was similar to that of the NC group (*p* > 0.05, Figure 4H). This result agreed with the report by Yue et al. (35).

At the genus level (Figure 3F), compared with the NC group, the relative abundance of *g_Akkermansia* (*p* < 0.01) and *g_Faecalibaculum* in the HC group was decreased, and the relative abundances of *g_Bacteroides* (*p* < 0.001) and *g_Alistipes* (*p* < 0.01) were significantly increased (Figures 4I–L). After PIDF supplementation, the relative abundance of *g_Akkermansia* and *g_Faecalibaculum* was improved in both the LIDF and the HIDF groups, although this was insignificant. Meanwhile, the relative abundance of *g_Alistipes* in LIDF and HIDF groups significantly increased by 2.68 times and 3.46 times, respectively (Figure 4K). At the same time, feeding a high-fat diet increased the relative abundance of *g_Bacteroides* (Figure 4L).

**Figure 5:**
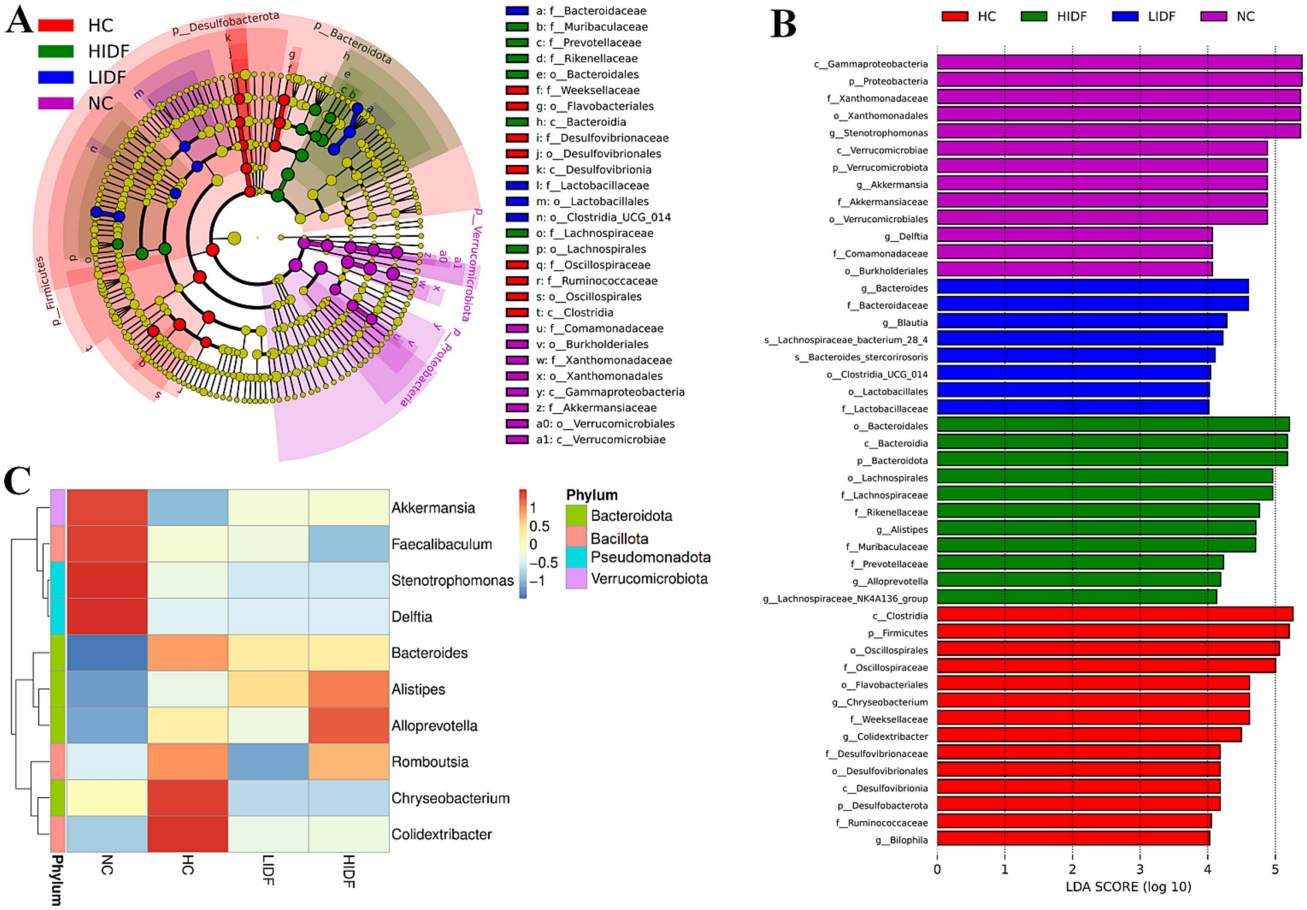
Regulation of PIDF on gut microbiota of high-fat diet mice. **(A)** LEfSe; **(B)** Linear discriminative analysis effect size analyses of statistically significant taxa; **(C)** Heat maps between the top 10 genera and physiological indexes.

Furthermore, linear discriminant analysis effect size (LEfSe) was used to analyze the typical bacterial groups with rich differences in the four groups of mice and to explore the influence of PIDF supplementation on the characteristic strains, and the results are shown in Figures 5A,B. The results showed that 52 bacterial taxa, whose abundance was significantly affected by the high-fat diet and PIDF intervention, were selected as OTUs. In the NC group, the dominant bacterial taxa are *c*_*Gammaproteobacteria*, *p*_*Pseudomonadota*, *f*_*Xanthomonadaceae*, *o*_*Xanthomonadales*, and *g*_*Stenotrophomonas*. The HC group was distinguished by *c*_*Clostridia*, *p*_*Bacillota*, *o*_*Oscillospirales*, and *f*_*Oscillospiraceae*. However, the LIDF group had a high abundance of *g*_*Bacteroides*, *f*_*Bacteroidaceae*, *g*_*Blautia*, and *s*_*Lachnospiraceae*. The similarity is that *o*_*Bacteroidales*, *c*_*Bacteroidia*, *p*_*Bacteroidota*, *o*_*Lachnospirales*, and *f*_*Lachnospiraceae* were more enriched in the HIDF group. This result was consistent with the findings of Zhao et al. (19), which reported that flaxseed cake dietary fiber ameliorates the harm of a high-fat diet mainly by restoring the gut microbiota composition.

**Figure 6:**
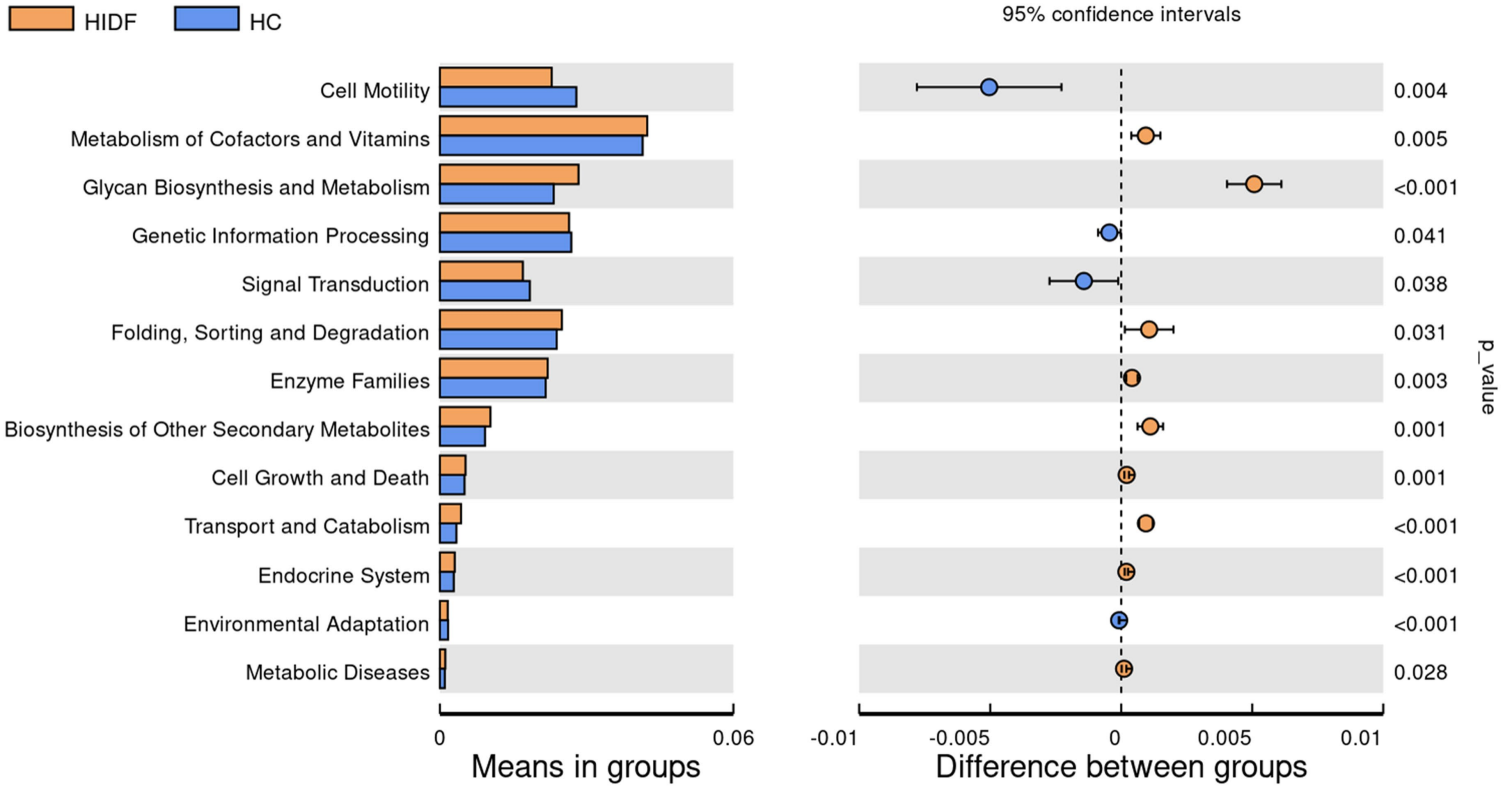
The functional prediction (PICRUSt) of the dominant gut microbiome.

Next, to explore the characteristics of the intestinal microbiome composition of mice in each group, correlation analysis was conducted between the top 10 bacterial taxa at the genus level and environmental factors. The results were visualized in the heat map (Figure 5C). The data showed that the relative abundance of *g_Colidextribacter*, *g_Chryseobacterium*, *g_Romboutsia*, and *g_Bacteroides* in the HC group increased due to a high-fat diet. In addition, the bacterial taxa of the LIDF and HIDF groups decreased to different degrees due to the treatment of PIDF. The functional prediction (PICRUSt) of the dominant gut microbiome was determined, and the functional composition of the intestinal microbiota at level 2 was shown in Figure 6. Compared with the HC group, the intervention of high-dose PIDF significantly changed 13 pathways, including 9 up-regulated pathways and 4 down-regulated pathways. The result indicated that high-dose PIDF supplementation significantly increased the abundance of Metabolism of Cofactors and Vitamins, Folding, Sorting and Degradation, Enzyme Families, Biosynthesis of Other Secondary Metabolites, Transport and Catabolism than that of the HC group, which is beneficial to lipid metabolism.

### 3.8 The effects on mouse caecum contents metabolic profiles

**Figure 7:**
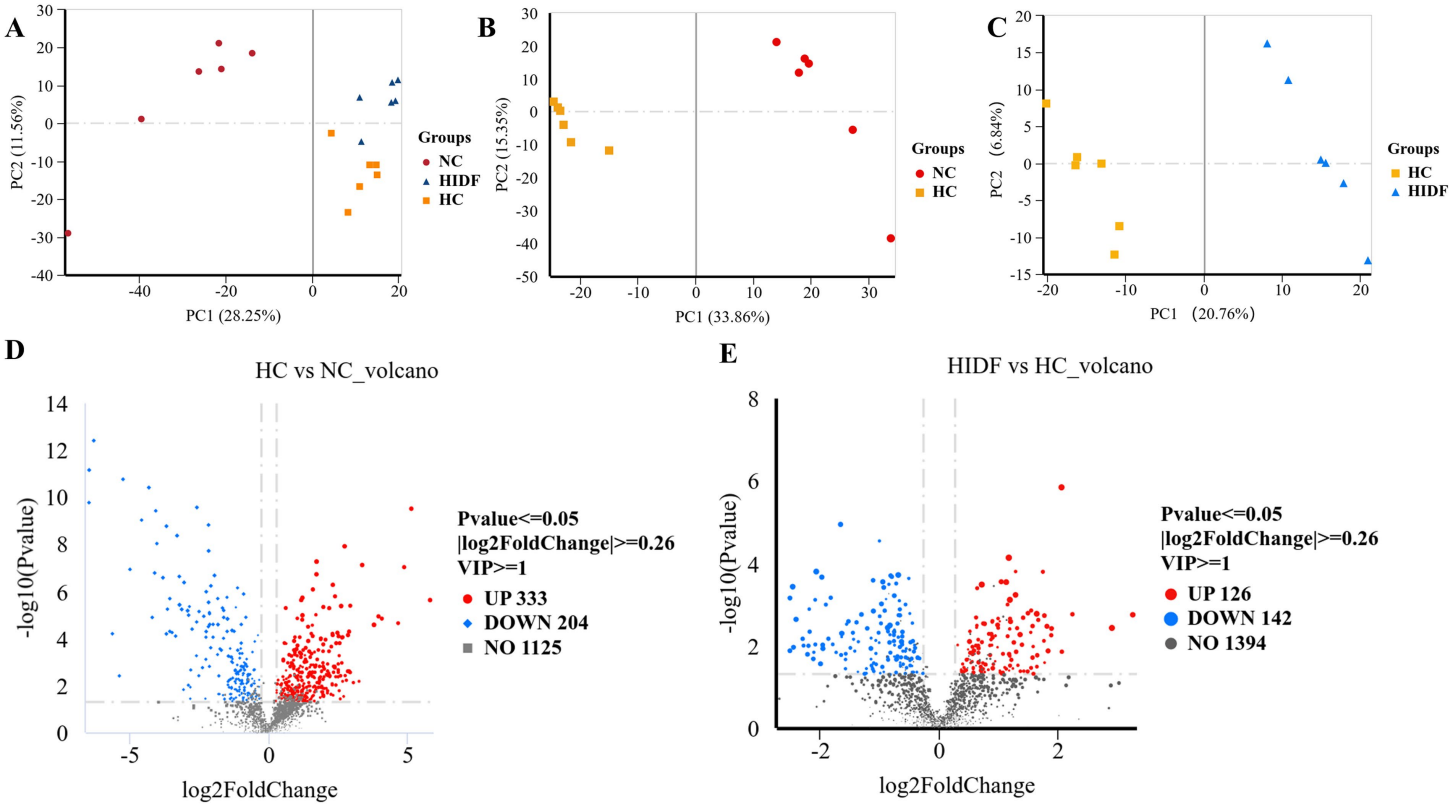
Effects of PIDF on metabolite profiles in high-fat diet mice. **(A)** PCA; **(B)** PLS-DA between HC and NC; **(C)** PLS-DA between HIDF and HC; **(D)** Volcano plot between HC and NC; **(E)** Volcano plot between HIDF and HC.

The cecum contents of mice were analyzed using non-targeted metabolomics by LC–MS/MS. The substances detected in the positive and negative ion scans were used for further analysis. PCA analysis was performed for the three groups (NC, HC, and HIDF), and the three groups were clustered separately, as depicted in Figure 7A. In the PLS-DA results, the HC and NC groups (Figure 7B) and the HIDF and HC groups (Figure 7C) were clearly separated, confirming the PCA findings. This showed that PIDF significantly altered the metabolites of high-fat-fed mice.

**Figure 8:**
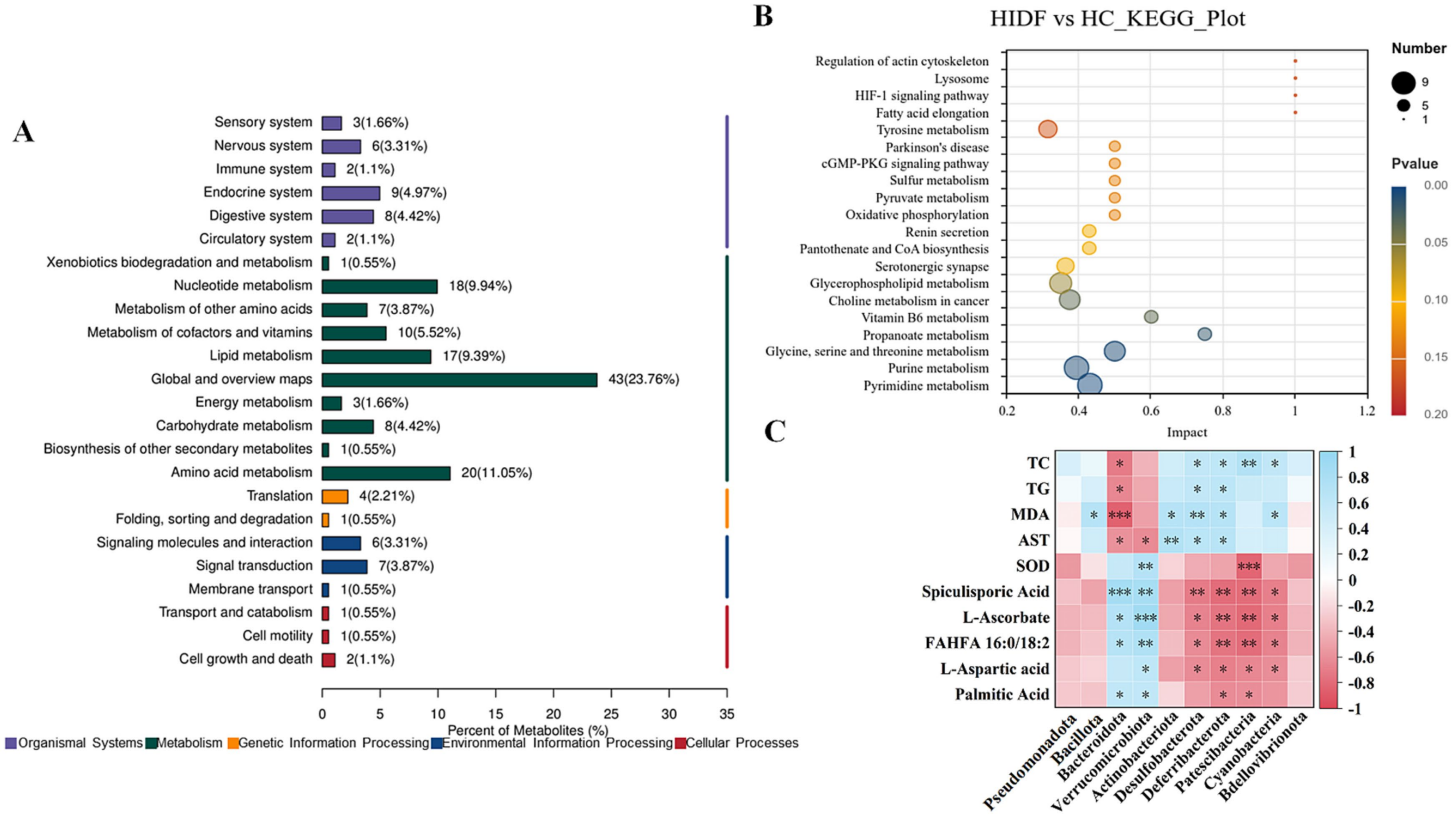
**(A)** KEGG topology; **(B)** KEGG pathway enrichment analysis; **(C)** Correlation between gut microbiota and its metabolites and hyperlipidemia.

A total of 1,662 metabolites (including those detected by positive and negative ion scanning) were identified based on VIP values greater than 1.0 and *p*-values less than 0.05. The results of the volcano plot showed that 333 metabolites were up-regulated, and 204 metabolites were down-regulated by the high-fat diet (Figure 7D). In the meantime, after the PIDF diet intervention, compared with the HC group, 126 metabolites were up-regulated, and 142 metabolites were down-regulated (Figure 7E). Based on the differential expression of metabolites in the HIDF and HC groups, we performed pathway analysis using the Kyoto Encyclopedia of Genes and Genomes (KEGG) topology. As seen from Figure 8A, lipid acid (spiculisporic acid, FAHFA 16:0/18:2, and palmitic acid), and amino acid (L-aspartic acid,) metabolism are the most significantly affected pathways. Further, MetaboAnalyst revealed that the PIDF diet led to the enrichment of pyrimidine metabolism, purine metabolism, glycine, serine, and threonine metabolism, as well as glycerophospholipid metabolism, as highly enriched metabolic pathways (Figure 8B). Therefore, these pathways may be key sites for PIDF to demonstrate its efficacy in reducing blood lipids.

### 3.9 Correlation between gut microbiota and metabolites and hyperlipidemia

To verify the effect of IDF on regulating intestinal microorganisms, Spearman analysis was used to study the correlation between key bacterial genera in feces and differential metabolites in cecum contents, and the results are shown in Figure 8C. Figure 8C showed that *p_Bacteroidota* and *p_Verrucomicrobiota* showed a significant correlation with 5 altered metabolites (spiculisporic acid, L-ascorbate, FAHFA 16:0/18:2, L-aspartic acid, and palmitic acid). Fatty acids play an essential role in regulating energy metabolism. Especially, *g_Bacteroidota* showed a significant correlation with hyperlipidemia indexes (TC, TG, MDA, and AST). In addition, the relative abundances of *g_Desulfobacterota*, *g_Deferribacterota*, *g_Patescibacteria*, and *g_Cyanobacteria* were correlated with some metabolites and hyperlipidemia indexes. The results showed that PIDF dietary supplements could regulate the gut microbiota and may influence its metabolites. This might be the reason why PIDF can alleviate hyperlipidemia caused by a high-fat diet.

## 4 Discussion

Hyperlipidemia is a disease with too much lipid in the blood, which can be induced by a high-energy diet (36, 37). *Polygonatum sibiricum* has excellent health benefits, such as hypoglycemic, hypolipidemic, regulating immunity, and so on (5). The activity study of the PIDF of *Polygonatum sibiricum* is not as extensive as those on polysaccharides and saponins. Hence, the high-fat diet-induced hyperlipidemia mouse model was used to research the anti-hyperlipidemic effect of PIDF (37). After 8 weeks of continuous PIDF supplementation, the dietary intake of mice in the low-dose and high-dose groups was significantly reduced. In our previous reports (15), it was indicated that PIDF exhibits strong oil-holding and water-holding properties, which increase the volume of food in the stomach and enhance satiety. Therefore, the body weights of mice in the LIDF and HIDF groups were significantly lower than those in the HC group. These results suggest that weight gain may be partially attributable to reduced energy intake. The result agreed with the report of Wang et al. (9), which revealed that okara’s IDF has an improving effect on hyperlipidemia in C57BL/6 J mice caused by a high-fat diet. In addition, the results of Chang et al. (38) showed that dietary supplementation with pear residue IDF slowed the weight gain caused by a high-fat diet, which agrees with this finding. In conclusion, the PIDF can alleviate excessive weight gain caused by sustained high energy intake.

As expected, PIDF supplementation significantly inhibited epididymal and perinephric fat weight in mice in the LIDF and HIDF. Consistent with the results of this study, Zhang et al. (39) reported that the IDF of soybean residue also showed a similar effect in alleviating fat accumulation in high-fat diet-fed mice. The same results reported by Chang et al. (38) indicated that in the high-fat diet group, adipocyte size decreased significantly after adding pear pomace’s IDF. In the meantime, the organ weights of mice in the LIDF and HIDF groups were significantly lower than those in the HC group. In the research by Fang et al. (24), *Auricularia polytricha* noodles supplemented in high-fat diet mice diets could significantly reduce liver weight accumulation and mitigate the development of fatty liver caused by a high-fat diet. In conclusion, the dietary intervention of PIDF can reduce fat accumulation and thereby alleviate metabolic diseases caused by excessive fat.

Hyperlipidemia often manifests as abnormal cholesterol metabolism and high lipid levels (40). Therefore, this study investigated the effects of PIDF on biochemical indices in high-fat diet mice. The results show that the TC and AST levels of the mice in the LIDF and HIDF groups were significantly lower than those of the HC group. In particular, the level of AST in the LIDF and HIDF was nearly identical to that of the NC group. AST was a standard detection indicator that evaluated liver function and detected liver lesions or damage in time (41). Meanwhile, TG, LDL-C, and GLU-YA levels in the HIDF were lower than those in the HC. In addition, the TG level of HIDF (1.08 mmol/L) was lower than that of pear pomace IDF (1.31 mmol/L) (38). The TC (4.01 mmol/L) and LDL-C (0.68 mmol/L) levels of HIDF were lower than those of soybean IDF (5.11 and 1.30 mmol/L, respectively) (9). These can be illustrated that PIDF has certain advantages in improving hyperlipidemia. While HDL-C is considered the good cholesterol in the human body, its increase benefits the body; conversely, a reduction in HDL-C content also falls under the category of dyslipidemia (42). The PIDF intervention significantly increased HDL-C in mice. These results suggest that PIDF can regulate cholesterol metabolism by binding to cholesterol and lowering cholesterol absorption. Those results were consistent with the previous reports. The study of Wang et al. (9) indicated that soybean insoluble dietary fiber plays a role in antihyperlipidemic effect by reducing TG, TC, and LDL-C levels and improving HDL-C levels in high-fat diet animals. Similarly, Liu et al. (32) demonstrated that dietary fiber from Ougan residue improved obesity caused by HFD, which was associated with a reduction in TC and TG levels and an increase in HDL-C levels in mice. In conclusion, dietary intake of PIDF can effectively alleviate symptoms of hyperlipidemia.

Oxidative stress is an adverse effect of free radicals in the body and is an essential factor in aging and disease (43). In the LIDF and HIDF, PIDF protects the liver from damage by reducing MDA levels, increasing SOD activity, and inhibiting fat accumulation in the liver. Consistent with previous reports, the mechanism by which polysaccharides from *Pleurotus eryngii* lower blood lipids also reflects a reduction in fatty liver and oxidative stress in high-fat diet mice (27). Meanwhile, the research conducted by Zhang et al. (11) emphasizes that IDF from brown seaweed *Laminaria japonica* can improve the morphology of liver cells in high-fat diet mice and play a significant protective role in liver injury. The current research results show that both low-dose (0.5 g/kg·BW) and high-dose (1.0 g/kg·BW) intake of PIDF has a good effect on improving hyperlipidemia. The World Health Organization suggests that adults should consume at least 25 g of dietary fiber every day. Additionally, the American Heart Association recommends that women consume approximately 25 g and men about 38 g of dietary fiber daily. Hence, the research dosage has certain potential implications for human nutrition.

The IDF can proceed directly to the cecum for fermentation without undergoing digestion. It is generally recognized that the community structure of gut microbial colonies plays a crucial role in regulating lipids (24, 33, 35). The impact of PIDF on the gut microbiome significantly reduces the *Bacillota* to *Bacteroidota* ratio. *Bacillota and Bacteroidota* played an essential role in regulating the metabolism of carbohydrates, bile acids, and lipids in the host (44). *p_Bacillota* can absorb energy, intensify fat accumulation, and increase the risk of metabolic diseases such as obesity. *p_Bacteroidota* reduces cholesterol synthesis and is a kind of probiotic (45). It is reported that a high *Bacillota* to *Bacteroidota* ratio will promote high energy intake and cause metabolic diseases (10). It is worth noting that this finding is consistent with the study results showing lower food intake in the LIDF and HIDF groups of mice. Besides, the reduction in the *Bacillota* to *Bacteroidota* ratio in the PIDF-supplement groups may be closely related to the up-regulation of fatty acids, which are indispensable to the human body (18). Furthermore, the functional prediction results suggested that the microbial abundance related to Transport and Catabolism metabolism increased in the HIDF group. This might explain the reason why the TG, TC, GLU-A, and LDL-C levels were lower in the HIDF group.

In addition, the PIDF supplement significantly improves the abundance of beneficial bacteria in the gut, such as *p*_*Bacteroidota*, *f_Muribaculaceae*, *f_Lachnospiraceae*, *f_Peptostreptococcaceae*, *g_Akkermansia*, and *g_Alistipes. p_Bacteroidota* can ferment carbohydrates and biotransform bile acids and other steroids, producing nutrients and energy needed by the body, which mainly lowers blood lipids and maintains health. It is reported that the reason why *f*_*Lachnospiraceae* is considered a beneficial bacterium is mainly related to its participation in the production of fatty acids (20). The metabolic products of the cecum of mice contain a considerable amount of fatty acid substances (FAHFA 16:0/18:2 and palmitic acid), which might be related to the increase in the richness of the relative abundance of beneficial microbial colonies.

Moreover, *f_Peptostreptococcaceae* is another category of bacteria that helps curb obesity. The report confirms that the increased quantity of *g_Akkermansia* can improve intestinal barrier integrity and alleviate obesity symptoms (33). Additionally, according to the report by Zhang et al. (11), *g_Akkermansia* was the dominant strain in the high-dose brown seaweed *Laminaria japonica* IDF treatment group. Moreover, Tian et al. (46) studied the mechanism by which barley leaf IDF improves intestinal inflammation in mice. They found that barley leaf IDF supplementation regulated the gut microbiome of mice by increasing the relative abundance of *g_Alistipes* by 5.2-fold, which was consistent with the results of this study. Moreover, short-chain fatty acids can regulate the immune response and inflammatory response of the body (47). On the one hand, *p_Bacteroidota* reduce cholesterol synthesis; *f_Lachnospiraceae*, *f_Peptostreptococcaceae*, and *g_Alistipes* could help reduce low-grade chronic inflammation in the body. On the other hand, *g_Akkermansia* could improve the integrity of the intestinal barrier. In conclusion, the beneficial bacteria play a synergistic role in the process of improving hyperlipidemia.

Meanwhile, the PIDF supplement significantly reduced the relative abundance of *f_Erysipelotrichaceae* and *f_Desulfovibrionaceae*, two types of bacteria closely associated with obesity and considered harmful (48, 49). Research indicates that *f_Desulfovibrionaceae* can increase the level of gut-derived lipopolysaccharides, which could cause damage to the integrity of the intestinal barrier, resulting in chronic inflammation and associated metabolic disorders (50). The results of Zhao et al. (19) showed that flaxseed dietary fiber also reduced the growth of these two types of harmful bacteria in the gut of mice on a high-fat diet. Hence, the intervention of PIDF can maintain the integrity of the intestinal barrier.

Furthermore, the results of functional prediction of the dominant gut microbiome indicated that relative abundance related to Metabolism of Cofactors and Vitamins was significantly increased (*p* < 0.01). Meanwhile, L-Ascorbate level in the metabolites of mice in the HIDF group was relatively higher and was proportional to the *p_Bacteroidota* and *p_Verrucomicrobiota*. In addition, in the HIDF group, Glycan Biosynthesis and Metabolism were extremely significantly up-regulated pathways (*p* < 0.001). Glycan metabolism may produce fatty acids, which may explain the increase in fatty acid substances (spiculisporic acid, FAHFA 16:0/18:2, and palmitic acid) in metabolites. In conclusion, the PIDF intervention treatment can regulate the microbial community structure of hyperlipidemic mice, improve intestinal barrier integrity caused by a high-fat diet, and thereby alleviate the symptoms of hyperlipidemia.

The results of the caecum contents metabolic profiles revealed that PIDF supplements improved the metabolism of lipids, amino acids, and glycerophospholipids, which alleviated the abnormal blood lipid metabolism caused by a high-fat diet. It has been reported that lipid metabolism is related to the PI3K/Akt signaling pathway. Once this signaling pathway is activated, it can promote the synthesis of fatty acids and the storage of triglycerides while inhibiting fat breakdown (51). In addition, Cho et al. (52) mentioned that enhanced glycerophospholipid metabolism can improve lipase activity and inhibit lipid metabolism abnormalities. Amino acid metabolism is closely related to glycolipid metabolism, which can affect the synthesis and decomposition of triglycerides by regulating the activity of enzymes (ACC and FAS) related to lipid metabolism and signaling pathways (such as mTORC1) (53, 54). Obesity and other metabolic diseases are affected by the amino acid metabolism, which will produce various metabolic products, such as SCFAs, branched-chain fatty acids, and other substances. Furthermore, amino acid metabolism can accelerate the tricarboxylic acid cycle, increasing energy consumption (55). In conclusion, the correlation analysis between gut microbiota and metabolites and hyperlipidemia indicated that PIDF supplementation increased the relative abundance of beneficial microorganisms and promoted the metabolism of lipids, amino acids, and glycerophospholipids.

## 5 Conclusion

The results of this study suggested that the improvement of lipid metabolism in mice induced by a high-fat diet with PIDF is associated with the regulation of the gut microbiota. The levels of TC, TG, and LDL-C in PIDF-supplemented mice were significantly reduced, while the levels of HDL-C were increased. Additionally, oxidative damage in the liver was alleviated, the *Bacillota* to *Bacteroidota* ratio of intestinal bacteria was decreased, and the abundance of beneficial bacteria was increased. In addition, the results of the correlation analysis showed that PIDF supplementation stimulated gut microbes, which may be the mechanism of action of PIDF in reducing blood lipids. However, the interaction between PIDF and gut microbes requires further study. In addition, the absence of dietary fiber from other sources is a limitation of this study. The results suggest that PIDF, as a low-fermentation dietary fiber, alleviates hyperlipidemia induced by a high-fat diet by reshaping the gut microbiota and regulating its metabolites. Therefore, these findings can provide a basis for applying PIDF in developing functional foods.

## Data Availability

The raw data supporting the conclusions of this article will be made available by the authors, without undue reservation.

## Footnotes

^1^ www.novogene.cn
