# Supplementary material for: The improvement effect of insoluble dietary fiber of Polygonatum sibiricum on hyperlipidemia in high-fat diet mice via gut microbiota and metabolites
Source: Front Nutr. 2025 Aug 4;12:1601867. doi: 10.3389/fnut.2025.1601867 (PMC12358496; doi:10.3389/fnut.2025.1601867)
Supplement: Supplementary file 1 [file Table_1.DOCX]

Supplementary Material

Table caption:

Table S1 Ingredients of high-fat diet feed.

Table S1

| Class | Ingredients | Mass ratio  (gm%) | Energy supply ratio (kcal%) |
| --- | --- | --- | --- |
| Protein | Casein | 200 | 800 |
|  | L-Cystine | 3 | 12 |
| Carbohydrate | Maltodextrin | 125 | 500 |
|  | Sucrose | 68.8 | 275 |
| Fiber | Cellulose | 50 | 0 |
| Fat | Soybean Oil | 25 | 225 |
|  | Lard | 245 | 2205 |
| Mineral | Mineral Mix S10026 | 10 | 0 |
|  | DiCalcium Phosphate | 13 | 0 |
|  | Calcium Carbonate | 5.5 | 0 |
|  | Potassium Citrate, 1 H20 | 16.5 | 0 |
| Vitamin | Vitamin Mix V10001 | 10 | 40 |
|  | Choline Bitartrate | 2 | 0 |
| Total |  | 773.85 | 4057 |
